# A simplified measure of nutritional empowerment: Using machine learning to abbreviate the Women’s Empowerment in Nutrition Index (WENI)

**DOI:** 10.1016/j.worlddev.2022.105860

**Published:** 2022-06

**Authors:** Shree Saha, Sudha Narayanan

**Affiliations:** a Dyson School of Applied Economics and Management, Cornell University, Ithaca, NY, United States; b International Food Policy Research Institute, South Asia Region, New Delhi, India

**Keywords:** Empowerment, Nutrition, Machine learning, LASSO, Gender, India, South Asia

## Abstract

Measuring empowerment is both complicated and time consuming. A number of recent efforts have focused on how to better measure this complex multidimensional concept such that it is easy to implement. In this paper, we use machine learning techniques, specifically LASSO, using survey data from five Indian states to abbreviate a recently developed measure of nutritional empowerment, the Women’s Empowerment in Nutrition Index (WENI) that has 33 distinct indicators. Our preferred Abridged Women’s Empowerment in Nutrition Index (A-WENI) consists of 20 indicators. We validate the A-WENI via a field survey from a new context, the western Indian state of Maharashtra. We find that the 20-indicator A-WENI is both capable of reproducing well the empowerment scores and status generated by the 33-indicator WENI and predicting nutritional outcomes such as BMI and dietary diversity. Using this index, we find that in our Maharashtra sample, on average, only 35.9% of mothers of children under the age of 5 years are nutritionally empowered, whereas 77.2% of their spouses are nutritionally empowered. We also find that only 14.6% of the elderly women are nutritionally empowered. These estimates are broadly consistent with those based on the 33-indicator WENI. The A-WENI will reduce the time burden on respondents and can be incorporated in any general purpose survey conducted in rural contexts. Many of the indicators in A-WENI are often collected routinely in contemporary household surveys. Hence, capturing nutritional empowerment does not entail significant additional burden. Developing A-WENI can thus aid in an expansion of efforts to measure nutritional empowerment; this is key to understanding better the barriers and challenges women face and help identify ways in which women can improve their nutritional well-being in meaningful ways.

## Highlights

• We develop simplified indicator of nutritional empowerment the Abridged Women’s Empowerment in Nutrition Index (A-WENI).
• We use machine learning techniques (LASSO) to reduce the number of indicators in the index from 33 to 20.
• A-WENI is developed for a five-state survey in India and validated with new data from the Indian state of Maharashtra.
• A-WENI reproduces well the empowerment status based on WENI and like WENI is a good predictor of nutritional outcomes.
• A-WENI can be more easily incorporated into general purpose rural surveys to capture women’s empowerment and nutrition.

## 1 Introduction

The United Nations has identified the achievement of gender equality and empowerment of all women and girls as one of the Sustainable Development Goals. While the notion of equality is somewhat straightforward and perhaps better understood, empowerment is harder to define. A widely accepted definition is based on Kabeer (1999), who characterizes empowerment as an individual’s capacity to make strategic choices in life in a context where this ability was previously denied to them (pg. 19). Empowerment is often conceptualized as encompassing a number of different aspects such as agency, autonomy, self-direction, self-determination, liberation (Ibrahim and Alkire, 2007, Malapit et al., 2019, Kabeer, 1999). An individual’s agency itself refers to multiple aspects including, for instance, intrinsic agency (power within), instrumental agency (power to) as well as collective agency (power with) and influence (power over). Others emphasize that the opportunity structure faced by individuals and an individual’s access to resources are key elements of empowerment (Kabeer, 1999, Alsop and Heinsohn, 2005). Empowerment is thus complex, dynamic and multidimensional. While there is no consensus on a single definition of empowerment, there is currently a significant congruence of views on what it represents. For example, most agree that empowerment is multidimensional and includes not just agency but also the terms on which resources can be accessed and norms that govern such access as well as knowledge of these. There is also agreement that it is a process and empowerment in some spheres can occur alongside disempowerment in other spheres. It is important too to distinguish empowerment in specific realms, economic, health and nutrition, for example, from a generalized notion of empowerment.

While empowerment is desirable for its own sake, it can also have instrumental value in forwarding the well-being of individuals, especially crucial for women, both in terms of economic and non-economic attributes such as health and nutrition (Kadiyala, Harris, Headey, Yosef, & Gillespie, 2014). Economists, for instance, note that although economic development and women’s empowerment go hand in hand and that development typically leads to empowerment, it is important nevertheless to design policy explicitly for empowering women (Duflo, 2012). The call for gender equality and empowerment of all women and girls is therefore a call for policy action geared explicitly towards empowering women and girls.

Yet, our ability to track progress on women’s empowerment, to examine its relationship with women’s wellbeing and to assess the empowering effect of public policy depends crucially on our ability to reliably measure empowerment (Alsop and Heinsohn, 2005, Malhotra et al., 2005) and that uses data that are easy to collect. In this paper, we outline one such effort to create a measure of empowerment in the domain of nutrition. Building on prior work in the area of nutritional empowerment by a team of interdisciplinary researchers, discussed later, we focus on creating a leaner version of the Women’s Empowerment in Nutrition Index (WENI) using machine learning techniques that can be incorporated in general purpose surveys without difficulty.

Current evidence suggests that there is a large number of measures that vary based on the concept or definition of empowerment, the domain in which empowerment is studied (economic empowerment, empowerment in agriculture, empowerment in livestock, nutritional empowerment, etc.), methods used in the selection and aggregation of indicators and data used (Pratley, 2016, Laszlo and Grantham, 2017, Pereznieto and Taylor, 2014, Santoso et al., 2019). Reviews note for example that there are over 40 measures for economic empowerment and as many as 181 measures for empowerment relating to health and nutrition (Pratley, 2016, Laszlo and Grantham, 2017). Further, critics argue that many of these measures are not sufficiently well-grounded in theory. Many are based solely on data reduction techniques that often select one variable at the expense of others because they are all correlated, although they represent very different aspects of empowerment. Still other measures are not comprehensive enough for the purpose it is supposed to serve (Richardson, 2018, Malhotra et al., 2005, Heckert and Fabic, 2013). Apart from debates on how best to measure empowerment, a related challenge in measuring empowerment is that, owing to its complexity, it is typically cumbersome to implement (Alsop & Heinsohn, 2005). This often deters efforts to incorporate empowerment measures in general surveys of individuals and households, which capture instead just a few proxy indicators of empowerment.

There have been several recent attempts to develop simplified (sets of) indicators of empowerment. Ibrahim and Alkire (2007), for example, have focused on identifying an internationally comparable set of indicators that are relatively easy to collect. Others have created useful libraries of specific survey questions that can elicit information on different dimensions of empowerment (Laszlo and Grantham, 2017, Glennerster et al., 2018). Still others have tested whether the measures of empowerment are appropriate for the context and captures the underlying concepts well (Hannan et al., 2020, Yount et al., 2019). Somewhat differently, developers of the Women’s Empowerment in Agriculture Index (WEAI), an important and widely used index for rural communities, have designed a more user-friendly Abbreviated Women’s Empowerment in Agriculture Index (A-WEAI) that reduces interview time by 30% as compared with the WEAI (Alkire et al., 2013, Malapit et al., 2019, Malapit et al., 2017).

This paper contributes to these efforts, responding to the need to develop leaner indices of empowerment that reduce the time and cost burden associated with data collection, especially in resource-constrained settings. Our focus is a specific measure of empowerment, the Women’s Empowerment in Nutrition Index (WENI). Developed by an interdisciplinary team of researchers, the WENI aims to capture empowerment in the realm of nutrition in rural contexts (Narayanan, Lentz, Fontana, De, & Kulkarni, 2019). The WENI, in its original form, was developed in the context of rural south Asia and comprises 33 indicators collected via a special purpose survey. The 33-indicator WENI relies on 59 different survey questions and is therefore potentially burdensome to respondents. Our effort in this paper is to develop a leaner version of this index with fewer indicators that can be more easily incorporated in general purpose household surveys for rural contexts. Our effort is motivated by the data gaps in current large-scale surveys that capture neither women’s nutrition nor their empowerment status particularly well. An abridged WENI (henceforth A-WENI) in our view would contribute to filling this gap.^1^

The central challenge in abbreviating any index is to be able to simplify the measure without compromising on either the richness or the spirit of the original index and to achieve this balance with minimal procedural subjectivity. As Richardson (2018) cautions, data reduction techniques can often conflate conceptually distinct indicators merely because they are correlated and this can undermine the conceptual basis and richness of such indicators. In general, abbreviating an existing index involves selecting a subset of indicators in ways that do not affect the overall performance of the index (Malapit et al., 2019). Current efforts to minimize the number of indicators captured in an aggregate empowerment index typically exclude a subset of indicators that are highly correlated with other preferred indicators that are already in the index, using Principal Components Analysis, (Confirmatory) Factor Analysis and so on. In this paper, we propose the use of machine learning or statistical learning techniques, in particular, supervised learning techniques, as a reliable, objective approach to simplify complex, multidimensional, multi-indicator indices of empowerment.

The application of machine learning in the area of economics has expanded in recent years (Athey, 2019, Storm et al., 2019) In the field of international development, some have used it in the context of ensuring data quality in surveys (Shah et al., 2020). Many of the applications thus far have however focused on obtaining better estimates or predictions of poverty (McBride and Nichols, 2018, Afzal et al., 2015), economic growth (Basuchoudhary, Bang, & Sen, 2017) or food insecurity (Lentz, Michelson, Baylis, & Zhou, 2019). As a data reduction tool for generating measures of welfare, Kshirsagar, Wieczorek, Ramanathan, & Wells (2017) apply LASSO techniques to identify variables that go into an index to be able to identify the poor and hence facilitate program targeting. Machine learning approaches have so far not been explored in the context of complex multidimensional indicators such as empowerment and our current work is among the first to demonstrate their potential (Saha & Narayanan, 2020). More recent efforts include combining machine learning techniques and qualitative methods to identify survey questions to measure specific aspects of empowerment such as agency (Jayachandran, Biradavolu, & Cooper, 2021).

Drawing on data used to create the 33-indicator WENI, we apply LASSO (least absolute shrinkage and selection operator), a popular machine learning technique to identify the candidate constituent indicators of A-WENI. We then validate it afresh out of sample, implementing a survey in rural Maharashtra, India, for this purpose. Our findings suggest that the WENI can be abridged to 20 indicators, while remaining faithful to the concept of nutritional empowerment and still reproduce well the nutritional empowerment scores and status based on the 33-indicator WENI. Whereas the 33 indicators of WENI involve 59 unique questions in the survey, the A-WENI requires only 28 questions to elicit its 20 constituent indicators. We believe that the 20 indicators in A-WENI represent significant reduction in survey time, ranging from 20 to 30 min. Further, the A-WENI remains a good predictor of nutritional status of individuals, including BMI and dietary diversity scores, just like the original WENI. In this paper, we detail the process by which machine learning techniques can contribute to developing sophisticated yet simple measures of empowerment, based on more complex preexisting measures and present our findings from the validation of A-WENI in rural Maharashtra.

This paper is organized as follows. In Section 2, we describe in detail the WENI as it was originally developed. In Section 3 we elaborate on the machine learning technique we propose to use and then apply it to identify, develop and validate a simplified measure of nutritional empowerment, i.e. A-WENI, using the following steps. We first use survey data from five Indian states (the WENI Survey) and partition these into a training and a validation set. We implement LASSO on the training set to select candidate indicators for A-WENI. We then validate A-WENI using the validation set to arrive at a final set of indicators for A-WENI. We compute both WENI and A-WENI for the WENI Survey. We compare nutritional empowerment scores and status predicted by the two and test whether A-WENI, like its parent index WENI, is also a significant predictor of nutritional outcomes. In Section 4, we validate A-WENI with new survey data from Maharashtra (A-WENI Survey); for these new data, we compare WENI and A-WENI, the nutritional empowerment scores and status based on each of them and examine if A-WENI is a significant predictor of nutritional outcomes. This process enables us to validate A-WENI for a new dataset from a new context. Section 5 deals with robustness checks and caveats, notably that the A-WENI has been developed for the South Asian context and needs further validation in other potentially different contexts. Section 6 concludes the discussion, highlighting the potential usefulness of A-WENI.

## 2 The Women’s Empowerment in Nutrition Index (WENI)

The Women’s Empowerment in Nutrition Index (WENI) was created in response to a perceived need for a measure of empowerment that is salient to women’s nutritional well-being, as opposed to generic measures of empowerment that don’t often pertain to nutrition or correlate well with nutritional status (Narayanan et al., 2019). The effort also explicitly shifts the focus of the relationship of women’s empowerment to their own nutritional status rather than that of their children.

The WENI project focused on conceptualizing nutritional empowerment and then developing and validating WENI specifically for the rural South Asian context. Through participatory qualitative approaches and desk review of literature on empowerment and nutrition, the authors identify key interdependent factors and pathways that can enable better nutrition for women (Narayanan, Lentz, Fontana, & Kulkarni, 2022). Further, they focus on situations of scarcity and deprivation, where a woman’s knowledge and ability to act to secure food might make a difference to her nutritional status. In other words, whereas women can attain adequate nutritional outcomes if their socio-economic status is high, the underlying premise of nutritional empowerment is that it matters if nutritional outcomes reflect a meaningful choice or not.

Accordingly, nutritional empowerment is defined as “the process by which individuals acquire the capacity to be well fed and healthy, in a context where this capacity was previously denied to them” (p. 2 Narayanan et al., 2019). This process entails “ acquiring knowledge about and say over nutritional and health practices; gaining access to and control over intake of adequate and nutritious food; and being able to draw support from both family and other institutions to secure and maintain an adequate diet and health” (p. 2 Narayanan et al., 2019). This process, they note, implies changes not just at the level of individual, or their ability to act but at the level of broad structures constraining a fairer distribution of resources and power (Narayanan, Lentz, Fontana, & Kulkarni, 2022).

### 2.1 The WEN grid

To operationalize this conceptualization of nutritional empowerment, the WENI researchers amalgamate insights from two distinct streams of literature – the literature on women’s empowerment, drawing heavily on Kabeer (1999), including four dimensions – knowledge, resources, agency and achievements – and the literature on the drivers of nutrition following the UNICEF (1990) framework for child nutrition and identifying three domains – food, health and institutions – that are salient for women’s nutrition.^2^

Narayanan et al. (2019) and Narayanan, Lentz, Fontana, & Kulkarni (2022) then propose a WEN Grid to organize the domains and dimensions into a matrix, organized under the three domains of food, health and institutions and key dimensions of empowerment for nutrition: knowledge, resources, and agency that lead to achievements in terms of nutritional outcomes. The WEN Grid serves the basis of identification of factors that constitute nutritional empowerment. The authors advocate many applications of the WEN Grid – as a heuristic device to conduct nutrition and empowerment diagnostics, as a scorecard to identify the magnitude of the impediments to nutritional empowerment and as the basis for aggregating these scores into a catch-all measure, the Women’s Empowerment in Nutrition Index (WENI).

WENI is thus conceived of as a metric that aggregates measures of these factors into a single number and is described in detail below.^3^ In its original formulation, nutritional outcomes like BMI, anaemia and dietary diversity are considered (nutritional) achievements relating to nutritional empowerment but not explicitly a part of WENI itself. Nutritional achievements are therefore used only to validate WENI and to assess the associative strength between the metric representing nutritional empowerment and nutritional outcomes.

It is useful to note that the WENI seeks to capture the status of an individual at a particular point of time even though nutritional empowerment is conceptualized as a process. The idea is that WENI can be measured at different points of time, enabling us to track progress in eliminating the barriers to empowerment. Although the WENI focuses on women specifically, the empowerment measure created can be used to identify empowerment status of any adult.^4^

### 2.2 Computing WENI

The WENI is constructed using 33 indicators covering the seven Domain-Dimensions or DDs (Table 1 lists these). These 33 indicators straddle several themes and typically there are multiple indicators representing different aspects that capture a single theme, described in detail in subsequent paragraphs. To compute WENI, each indicator is first converted to a binary variable, where 1 represents being empowered with respect to the specific indicator and 0 otherwise. A detailed discussion of these are found in Narayanan et al. (2019). and are therefore not presented here. A score is then computed for each DD indicating the proportion of indicators on which the individual is deemed to be empowered for that DD. The DD-specific score thus ranges between 0 and 1. These DD-specific scores are then averaged over the seven DDs to generate the index scores, weighting each DD equally. This score, the WENI, thus ranges between 0 and 1 (both inclusive). A cut-off for the aggregated index, 0.5 in this case, is set and on the basis of the cut-off, individuals who have scores less than 0.5 are classified as nutritionally disempowered and those with scores above the cut-off are classified as empowered.^5^ Several issues around the sensitivity of the WENI to cutoffs and the form of indicators are addressed in Narayanan et al. (2019) and are beyond the scope of this paper. We do however test the sensitivity of A-WENI to different thresholds in the Supplementary Materials. The 33-indicator WENI was originally constructed and validated in five Indian states – Bihar, Odisha, Tamil Nadu, Kerala and West Bengal – using data collected in 2017–18 (henceforth, the WENI Survey).

**Table 1:** List of Indicators in WENI and their Description.

No.	Variable name	Themes	Variable Description
1	FAagrisay	Say in productive activities	Has some say in agricultral activities(=1)
2	FAmajminsayent		Atleast some say in major or minor household enterprise decisions(=1)
3	FAassearnconsentown	Control/say in income and expenditure	Earnings from asset owned by respondent has not been used without consent
4	FAcashcontrol		Has cash as independent source of money & some control over how to spend it(=1)
5	FAdecisionpaidwrkbin		Decision to undertake or not undertake paid work own (=1)
6	FAnorestorwillstop		Faces no food restriction or from own will or can stop when wants(=1)

7	FKcalcium		Knowledge on calcium
8	FKiodine		Knowledge on iodine

9	FReatlast	Eating norms	Eat last only rarely/sometimes/never(=1)
10	FRfinsupportagrihhenter	Access to support and to assets	Some financial support/aid in HH enterprise or agriculture(=1)
11	FRjobandpds		Someone in household has both jobcard and rationcard(=1)
12	FRland		Own land in your name
13	FRpaidwork	Participation in income generating activities	Does paid work as employee(=1)
14	FRselfemployment		Has farm/non farm own employment(=1)
15	FRsourceincomediversitybin		Atleast 3 diverse sources of income out of 5 (=1)
16	FRwrkoutalone		Social norms permit women to work outside the village, alone

17	HAalonefortreatment		Can go alone to health centre for treatment if need be(=1)
18	HAhdecideownhealth		Can make decision on own health(=1)
19	HAhealthvisitpermission		No expectation to take permission from family before visiting health centre(=1)

20	HKanemia		Knowledge on anemia
21	HKmalaria		Knowledge on malaria
22	HKors		Knowledge on ors

23	HRdrinktoivent	Access to improved water, sanitation and smoke free kitchen	Household has access to improved water & improved toilet & ventilation(=1)
24	HRhassistwhensick	Support when ill and health seeking	Get assistance in household chores when ill(=1)
25	HRhoursmktworkbin	Work/energy expenditure and working conditions	Marketable work (paid or hh enterprise) > 8 h (=1)
26	HRriskinjhealth		No risk of injury or major health problem in any activity(=1)
27	HRintensityany	Support in work	Does no paid/unpaid activity that is back breaking or heavy(=1)

28	Ianymemberownaccord		Member of any group with own accord(=1)
29	Idoveil		Never practice Ghonghat/Burkha/Pallu/Purdah(=1)
30	Ifreedommove		Has mobility to go to bank or post office or family alone(=1)
31	Imobileinformationgovt		Individual gets information on government schemes from mobile phone(=1)
32	Inoviolenceorsupport		Experiences no physical abuse or if does, has support within family (=1)
33	Iparticipatedany		Politically active in any activity in past 5 years(=1)

The variable prefixes stand for: FA-Food Agency; FK-Food Knowledge; FR- Food Resources;HA-Health Agency; HK-Health Knowledge; HR- Health Resources; I- Institution.

### 2.3 WENI Indicators and Themes

As mentioned earlier, WENI indicators for an individual span seven domain-dimensions or DDs – namely food-knowledge (FK), food-resources (FR), food-agency (FA), heath-knowledge (HK), health-resources (HR), health-agency (HA) and institutions (I). Recall that these are constructed by combining three domains (food, health and institutions) and three dimensions(knowledge, resources and agency). Narayanan et al. (2019) consider institutions as a separate domain incorporating factors such as legal rules, general community norms, not pertaining specifically to food, health or fertility that represent nutritional empowerment.

These indicators were chosen based on formative desk reviews and field-based qualitative research to represent salient themes that capture different aspects of nutritional empowerment (Narayanan et al., 2019). Table 1 presents the list of indicators used in constructing WENI and also gives the list of themes by DD.

The food and health knowledge (FK and HK, respectively) DDs consists of indicators measuring knowledge of nutrition and health. Food-agency is composed of two separate themes: say in productive activities and control over income and expenditure. The DDs of food-resources (FR) and health-resources (HR) comprises three and four themes respectively. Indicators are categorized under each of these themes. For example, in the health-resources (HR) DD, the theme “ Support when ill and health seeking ” is measured by whether an individual has sought treatment when ill and whether they get assistance when they are sick. The indicators in the domain of institutions (I) capture different social and legal norms and they range from whether the individuals are members of any group, whether they receive information about government schemes or faces restrictions on movement to the individual’s participation in public spheres (for example, speaking in public or contesting elections). In this paper, we maintain the classification of indicators into DDs and its themes as in the original WENI so that these are treated as inherited and fixed.

Based on these variables, a 33-indicator WENI (Table 1) can be computed for all individuals; the information is gathered via a special purpose survey that takes between 30 and 90 min to administer.^6^ Given the number of indicators to compute the index, the burden on respondent’s time (especially young mothers who are simultaneously involved in child care and domestic chores) is quite heavy. This is true of most empowerment surveys, for example the pilot 1.1 of WEAI took about 62 min in Bangladesh (Malapit et al., 2019, Malapit et al., 2017). The survey time and complex nature of data collected are likely serious deterrents to uptake and can result in the systematic exclusion of these types of measures from household surveys. Our attempt therefore is to remedy this problem and we use the 33-indicator WENI as a starting point for our effort to abridge the index.^7^

## 3 Designing an Abridged WENI (A-WENI)

Our aim is to create a leaner WENI with fewer indicators without compromising on its ability to reproduce the nutritional empowerment scores and empowerment status of the 33-indicator WENI, while also predicting nutritional outcomes. The survey module of such an abridged index can then be more conveniently incorporated into a general purpose survey for rural communities and help measure nutritional empowerment with few additional resources.

The process of eliminating some indicators rather than others however needs to have a sound basis and as far as possible, devoid of subjectivity. At the same time, it should remain faithful to the normative rationale for the choice of these indicators and consistent with the original 33-indicator WENI. Our goal is therefore to identify the subset of indicators that best predicts the nutritional empowerment scores and status based on the 33-indicator WENI. This is in contrast to, for instance, identifying the subset of indicators that best predicts nutritional outcomes. Thus, as with the original WENI, nutritional outcomes do not factor in explicitly in the designing the index and is used only for the purpose of validation. We use machine learning techniques, elaborated in the next section, to identify a subset of indicators that will comprise the A-WENI, using nutritional empowerment scores based on the 33-indicator WENI as the response variable. The abridged set of indicators then undergoes several sensitivity analyses and validation tests on the data from the WENI Survey.

Once a plausible A-WENI is identified, we then validate it afresh with new data from Nashik, Maharashtra in western India (henceforth the A-WENI Survey). Our proposed methodology consists of the following steps:

1. Indicator selection using the five-state WENI Survey based on a set of predetermined criteria,
2. Constructing A-WENI using the selected indicator list for the five-state WENI Survey
3. Comparing performance of the reduced set of indicators and validating A-WENI using the five-state WENI Survey,
4. Validating A-WENI for new data from the Maharashtra A-WENI Survey.

The following sections discuss each of these steps in detail.

### 3.1 Indicator selection using LASSO techniques

#### 3.1.1 An overview of LASSO techniques

While there are several ways to reduce the number of indicators or variables such as principal components, factor analysis, backward and forward regression, we use the concept of supervised machine learning algorithms. Though this technique is used frequently in computer sciences, genome studies and financial markets, it has not been used much in the field of development, until recently.

In this paper we use machine learning techniques as a dimensionality reduction/ feature selection tool. Usually, machine learning techniques can be classified broadly into two categories – unsupervised learning and supervised learning. The former tries to uncover the data structure based on association and classification, without any prior knowledge of the data. The latter, in contrast, predicts the outcome based on existing data (Wuest et al., 2016, Schrider and Kern, 2018). In short, unsupervised techniques are data analysis approaches that identify correlations between variables without a dependent variable; supervised techniques are those that explore correlations between a set of variables that are used to predict a dependent variable. Popular statistical techniques such as Principal Component Analysis, Clustering etc. are considered unsupervised machine learning because they explore relationships between covariates without explicitly attempting to predict outcomes (Schrider & Kern, 2018). The advantage of machine learning over other statistical methods arises from its accurate predictions, ability to deal with high-dimension data along with its ability to simulate data in the absence of actual data. Most importantly machine learning allows the use of data as they are “ in nature, rather than in a way we represent it in a model”(Schrider & Kern, 2018).^8^

Starting the analysis from a known dataset called the training data, in our case, a subsample of the WENI Survey, the algorithm creates an inferred function that then predicts the outcome/future events that generates the closest results. We can compare this output with the actual and modify the model accordingly to get better results or predictions. Once the model is trained adequately on the training data, it can predict an outcome for any new data input. Even though there are several supervised machine learning algorithms, we use a technique called LASSO (Least Absolute Shrinkage and Selection Operator) for the following reasons.^9^

Unlike other machine learning algorithms, LASSO can perform both variable selection and regularization in order to improve the predictability and interpretation of statistical models it produces (Tibshirani, 1996). The LASSO is similar to the classical Ordinary Least Squares (OLS) which minimizes the sum of squared deviations between observed and model predicted values, but additionally imposes a penalty if coefficients are far from zero (Ahrens, Hansen, & Schaffer, 2020) (Eq. 1). The LASSO thus minimizes the mean squared error subject to a penalty on the absolute size of coefficient estimates.

To implement LASSO, we estimate the following model on a sub-sample of the five-state WENI Survey data.

(1) β^Lasso(λ)=argmin1n∑i=1nnut_sci-xi′β2+λn∑j=1pφjβj

Here, $\mathrm{nut}\_{\mathrm{sc}}_{i}$ is the individual’s nutritional empowerment score and $X_{i}$ is the vector of 33 indicators. The tuning parameter λ controls the penalty level and $\varphi_{j}$ are predictor-specific-penalty loadings. Supervised learning allows us therefore to reproduce the nutritional empowerment scores constructed on the basis of the 33-indicator WENI. We choose to use nutritional empowerment scores (WENI) as the variable to be predicted, given the prevailing argument that an empowerment status derived on the basis of these scores involves imposing thresholds that might be arbitrary (Richardson, 2018). An alternative approach where we predict the nutritional empowerment status is presented where appropriate to illustrate specific points.

Unlike the OLS, however, the LASSO imposes a penalty on the absolute size of coefficients estimates. It shrinks some coefficients and sets others to zero (Tibshirani, 1996, Ahrens et al., 2020). This way LASSO reduces model complexity and assists in feature selection, while keeping all predictors in the model. This feature of LASSO is contrast with forward/backward selection models where we are unable to find the impact of the removed variable on the outcome. However, like all regularized regression methods, LASSO too relies on tuning/penalty parameters that control the degree of penalization. In this case, there are three approaches to choosing the penalty level (λ); we discuss briefly each of these methods in the following paragraphs. We use all three in our analysis. Our choice of λ determines the number of indicators that will comprise A-WENI and our use of the various approaches allows us to examine the range of sets of candidate indicators.

• *Data driven approach (CVLasso)*: This is the classical approach using cross-validation method of re-sampling data to optimize out-of-sample prediction performance. Also known as the *k*-fold cross validation, this method involves partitioning a dataset into approximately equal *k*-folds and estimating the model on all modules (training set) except the $k^{\mathrm{th}}$-fold which is treated as the validation set. Predictive performance for a range of λs is assessed using the validation data. This process is repeated till all modules (thereby all data points) have been used for validation once. However, since the model is estimated *k* times, this approach is computationally intensive and time consuming. This method is useful for small datasets which are difficult to partition into testing and validation sets.
• *Information criterion approach (LASSO with IC)*: According to this approach, typically the selection of λ can be made using different information criteria like the Akaike Information Criterion (AIC), Corrected Akaike Information Criterion (AICC), Bayesian Information Criterion (BIC) and Extended Bayesian Information Criterion (EBIC). Though the computation of the information criteria is easy, data-driven, and its theoretical properties well known, they are less robust to violations of independence and homoscedasticity assumptions.^10^
• *Theory driven approach (RLasso)- “Rigorous” penalization*: The name rigorous penalization is rooted in its strong theoretical framework. This approach requires three conditions to be satisfied to guarantee that a LASSO is consistent in terms of prediction and parameter estimation. Rigorous lasso provides the additional benefits of dealing with heteroscedasticity and places high priority on controlling over-fitting and thus produces very parsimonious models. But given the focus on controlling overfitting, the cross-validation model may outperform in terms of prediction tasks.^11^

#### 3.1.2 Selecting indicators for A-WENI

We implement LASSO using data from the five-state WENI Survey data that were used to construct and validate the original 33-indicator WENI. We use data from two states, Bihar and Odisha consisting of 971 individuals as our training dataset and use data from three other status, Tamil Nadu, Kerala and West Bengal (1427 individuals) as our validation set. We choose Bihar and Odisha as the training sites because the WENI was first developed for these two states (Narayanan et al., 2019). The 33-indicator WENI was constructed for this subsample based on qualitative research, participatory methods and desk reviews that identified 168 candidate indicators. The WENI was then validated in TN, Kerala and West Bengal, where the 33-indicator WENI was used. In the current design, the fact that the training sites are dramatically different in terms of women’s empowerment from the testing sites, as the validation set, strengthens the case for the A-WENI’s external validity.

The original index is computed based on the procedure discussed in Section 2.2, using 33 indicators. The nutritional empowerment score so computed ranges from 0 to 1 and individuals are classified as nutritionally empowered if they have a score of 0.5 and above. Those who are nutritionally empowered are assigned 1 and those who are not are assigned a value of 0. The nutritional empowerment score that forms the basis of this 0–1 nutritional empowerment status serves as our response variable for LASSO. As mentioned earlier, we also use the binary nutritional empowerment status to verify that the two do not produce very different sets of indicators for A-WENI.

Using LASSO, we then identify a subset of indicators (from among the 33 indicators used to compute WENI) which ‘best’predicts the original nutritional empowerment score of individuals, as generated using the 33-indicator WENI. We use all three approaches of tuning the λ parameter. For each of these approaches, we compute both in-sample and out-of-sample Root-Mean Squared Error (RMSE). We choose the list of indicators that is identified by the method which has the lowest out-of-sample RMSE and in addition covers at least 50% of the themes from each DD. This additional criterion that indicators selected to be part of the A-WENI have to cover at least 50% of the themes in each DD, though subjective, aims to retain the spirit of WENI and prevents the index from being lopsided (Table 1 lists these ‘themes’ in each DD, which in turn are represented by indicators).^12^

As a first step, we estimate LASSO models based on default penalty parameters. The penalty level (λ) decides the number of indicators that get chosen. We find that using default penalty parameters, the indicator list for Rlasso, CVLasso contains 32 and 33 indicators respectively. While for Lasso with information criterion like AIC, AICC, BIC and EBIC the number of indicators chosen using the default penalty parameters is 33, 32, 28, 28 respectively (Table 2, Table 3).

**Table 2:** Performance of Indicators.

	RLasso	CVLasso	Lasso/aic	Lasso/aicc	Lasso/bic	Lasso/ebic	OLS
Default Indicator Performance							
No. of Selected regressors	32	33	33	32	28	28	33
No. Dropped regressors	1	0	0	1	5	5	0
In sample RMSE	0.01	0.00	0.00	0.01	0.03	0.03	0.00
Out of sample RMSE	0.03	0.00	0.01	0.02	0.05	0.05	0.00
Sample Size	971	971	971	971	971	971	971
							
20 Indicator List Performance							
No. of Selected regressors	20	20	20	20	20	20	–
No. Dropped regressors	13	13	13	13	13	13	–
In sample RMSE	0.04	0.04	0.04	0.04	0.04	0.04	–
Out of sample RMSE	0.07	0.07	0.07	0.07	0.07	0.07	–
Sample Size	971	971	971	971	971	971	–

Note: The training set is Bihar and Odisha. Out of sample RMSE is calculated on the three short survey states with a sample of 1427 individuals.

**Table 3:** Indicator List generated using different LASSO approaches on WENI survey data.

		Default Case		15-Indicator List		20 Indicator List (A-WENI)
Sl No	WENI	Rlasso	Lasso BIC/EBIC	RLasso	CVLasso/Lasso IC	CVLasso/Lasso-IC
1	FAagrisay	FAagrisay	FAagrisay			
2	FAassearnconsentown	FAassearnconsentown	FAassearnconsentown			FAassearnconsentown
3	FAcashcontrol	FAcashcontrol	FAcashcontrol	FAcashcontrol	FAcashcontrol	FAcashcontrol
4	FAdecisionpaidwrkbin	FAdecisionpaidwrkbin				
5	FAmajminsayent	FAmajminsayent	FAmajminsayent			FAmajminsayent
6	FAnorestorwillstop	FAnorestorwillstop	FAnorestorwillstop			
7	FKcalcium	FKcalcium	FKcalcium	FKcalcium	FKcalcium	FKcalcium
8	FKiodine	FKiodine	FKiodine	FKiodine	FKiodine	FKiodine
9	FReatlast	FReatlast	FReatlast	FReatlast		FReatlast
10	FRfinsupportagrihhenter	FRfinsupportagrihhenter	FRfinsupportagrihhenter			
11	FRjobandpds	FRjobandpds	FRjobandpds			
12	FRland	FRland	FRland			FRland
13	FRpaidwork					
14	FRselfemployment	FRselfemployment				
15	FRsourceincomediversitybin	FRsourceincomediversitybin	FRsourceincomediversitybin			
16	FRwrkoutalone	FRwrkoutalone				
17	HAalonefortreatment	HAalonefortreatment	HAalonefortreatment	HAalonefortreatment	HAalonefortreatment	HAalonefortreatment
18	HAhdecideownhealth	HAhdecideownhealth	HAhdecideownhealth	HAhdecideownhealth	HAhdecideownhealth	HAhdecideownhealth
19	HAhealthvisitpermission	HAhealthvisitpermission	HAhealthvisitpermission	HAhealthvisitpermission	HAhealthvisitpermission	HAhealthvisitpermission
20	HKanemia	HKanemia	HKanemia	HKanemia	HKanemia	HKanemia
21	HKmalaria	HKmalaria	HKmalaria	HKmalaria	HKmalaria	HKmalaria
22	HKors	HKors	HKors	HKors	HKors	HKors
23	HRdrinktoivent	HRdrinktoivent	HRdrinktoivent	HRdrinktoivent	HRdrinktoivent	HRdrinktoivent
24	HRhassistwhensick	HRhassistwhensick	HRhassistwhensick		HRhassistwhensick	HRhassistwhensick
25	HRhoursmktworkbin	HRhoursmktworkbin	HRhoursmktworkbin			
26	HRintensityany	HRintensityany				
27	HRriskinjhealth	HRriskinjhealth	HRriskinjhealth			
28	Ianymemberownaccord	Ianymemberownaccord	Ianymemberownaccord	Ianymemberownaccord	Ianymemberownaccord	Ianymemberownaccord
29	Idoveil	Idoveil	Idoveil	Idoveil	Idoveil	Idoveil
30	Ifreedommove	Ifreedommove	Ifreedommove	Ifreedommove	Ifreedommove	Ifreedommove
31	Imobileinformationgovt	Imobileinformationgovt	Imobileinformationgovt	Imobileinformationgovt		Imobileinformationgovt
32	Inoviolenceorsupport	Inoviolenceorsupport	Inoviolenceorsupport			
33	Iparticipatedany	Iparticipatedany	Iparticipatedany		Iparticipatedany	Iparticipatedany
						
Total Number of Indicators	33	32	28	15	15	20

Note: CVLasso and Lasso AIC Default chooses all indicators. Lasso AICC Default chooses all Indicators except FRpaidwork. RLasso 20 indicators have all the same as CVLasso and Lasso IC instead of FAmajminsayent the list has FRfinsupportagrihhenter.

Although the default mode fulfils our goal that the list of potential A-WENI candidates should contain at least half the number of indicators in each DD relative to the 33-indicator WENI (Table 1), we find that for most cases the number of indicators is quite large and hence does not serve our first goal of creating a lean index.

We therefore opt for a version of WENI with a target of populating it with 20 indicators. Arriving at 20 as the preferred number of A-WENI indicators involves judgement of what is the ideal balance between parsimony and faithfulness to the richness of a multidimensional index, a tradeoff we alluded to earlier. To do this we repeated the exercise, fixing the number of indicators at 25, 20, 15, and 10 and applying our judgement on whether the two goals of parsimony and richness are balanced adequately. Here we present the process for the 20-indicator list; the results for others are available from the authors.

To be able to generate an indicator list where the number of indicators is fixed, we adjust the penalty levels, as opposed to leaving it as the default levels. In this case, we adjust the penalty levels for all the three approaches such that all of them generate 20-indicator lists (Table 2). This type of approach of either fixing the penalty level to achieve a required number of variables or enforcing that a model always includes a certain variable is common. Kshirsagar et al. (2017), for example, demonstrates how the LASSO estimation can be programmed such that certain variables are always included in the model.^13^

We find that all the approaches generate the same set of indicators and covers at least 50% of the themes in each DD.^14^ Even though the indicator list is similar for all the three approaches and as such the choice of technique is rendered redundant in our analysis, we prefer the cross-validation LASSO technique, due to its more rigorous approach of choosing variables and its better performance (as compared with LASSO technique based on information criterion) in small datasets. Further, because the cross-validation under CVLasso uses subsets within the data, such that all data points have been used as a test data at least once, it is a preferred alternative.

Table 3 lists the indicators generated across the different approaches. Whereas the 33 indicators in the original WENI involved 59 unique questions is the survey, the A-WENI relies on only 28 questions to derive the 20 indicators in A-WENI. In this sense, although we did not explicitly use time or number of questions as criteria, our machine learning exercise appears to have identified indicators that require fewer distinct questions overall.

The abridged list of indicators draws on questions, many of which are already captured in several nationally representative surveys relating to health, such as the Indian Demographic and Health Survey – the National Family Health Survey (NFHS) or general purpose surveys such as the India Human Development Survey (IHDS). A detailed comparison of the overlap between these surveys is available from the authors. We note here that there remains a small list of indicators that feature in WENI, but not in the surveys we compare (3 indicators in IHDS-2 and 7 in NFHS-4) (Appendix Table A1). Given the substantial overlap of questions with other surveys, we expect the measurement of nutritional empowerment via A-WENI would entail little extra time or effort and can be incorporated in these surveys.

### 3.2 Computing A-WENI for the five-state WENI Survey

Our preferred candidate A-WENI consists of 20 indicators that by design is a subset of the 33 indicators used in constructing the original index (Table 1, Table 3). We now use the 20 indicators identified to compute A-WENI, using the same methodology for index construction as elaborated in Narayanan et al. (2019). Based on A-WENI, we identify those who are nutritionally empowered and those who are not and then validate A-WENI using the WENI Survey data to test its ability to predict nutritional outcomes, namely, BMI, anemia and dietary diversity.

### 3.3 Validating A-WENI using the five-state WENI Survey

The 20 indicator A-WENI must be validated within sample, before it can be used as a measure of nutritional empowerment. As mentioned earlier, the 20 indicators were generated for a subsample of the WENI Survey – Odisha and Bihar – as the training set. We now use the subsample of the WENI Survey from three other states – Kerala, Tamil Nadu and West Bengal, as the validation set. Validating the abridged index is a twofold process; first we test if the new index on an average classifies individuals as nutritionally empowered or disempowered ‘similar’ to the original. Second, we test whether the A-WENI is a good predictor of nutritional outcomes like the original index. The results of these two procedures will determine the usability of A-WENI as a substitute of the original 33-indicator WENI.

As mentioned above, the A-WENI would be a reliable proxy measure of nutritional empowerment only when it ranks individuals as empowered or disempowered in a way similar to the original WENI. To test this, we compute the rank order correlation between the abridged and original WENI nutritional empowerment index, which is a continuous score, and use Kendall’s tau-b to address issues related to ties in ranking. We find that the rank-order is preserved by A-WENI (Table 4). We also find a high positive correlation of 0.94 between the 33-indicator WENI and the A-WENI scores or index, i.e., the continuous variable on which the thresholds are imposed for identifying whether an individual is nutritionally empowered or disempowered.

**Table 4:** Area under the ROC curve and Rank order correlation between the abridged WENI and the original.

	Rlasso-15	CVLasso-15/ Lasso with IC-15	20 – Indicator List
ROC Curve			
Area under the ROC curve	0.83	0.84	0.90
Standard error	0.01	0.01	0.01
Lower bound	0.82	0.82	0.89
Upper bound	0.85	0.85	0.91
			
Rank Order			
tau-a	0.33	0.33	0.39
tau-b	0.68	0.71	0.80
p-value	0.00	0.00	0.00
Kendall score	949368.00	960510.00	1118760.00
se-score	28435.96	27717.43	28727.12
Observations	2398	2398	2398

Note: The sample is the existing data from 5 states.

To further test whether A-WENI classifies individuals correctly into nutritionally empowered and disempowered categories (based on the 33-indicator WENI), we conduct a Receiver operating characteristic (ROC) analysis (Table 4). The ROC analysis quantifies the accuracy of diagnostic tests used to discriminate between two states/conditions. This discriminatory accuracy of a diagnostic test is measured by its ability to correctly classify observations into their actual states/conditions. In this case, we examine if A-WENI correctly classifies an individual into being nutritionally empowered or disempowered as determined by the 33-indicator WENI. We find that 90% of the classifications into empowered or disempowered by the abridged WENI are correct (Table 4).

As part of the second procedure, we test whether A-WENI is a good predictor of nutritional outcomes using the validation set from the WENI Survey. We estimate a least squares (for continuous BMI, for the subsample who are not overweight or obese) and Probit regression model (for the binary variable), where normal BMI (between 18.5 and 25) is coded as one and underweight is coded as zero.^15^ Additionally, we also estimate a probit regression to examine if the nutritional empowerment leads to higher probability of achieving the minimum dietary diversity.^16^ We estimate the following models:

(2) $${\mathrm{BMI}}_{i}=\alpha_{0}+\beta_{0}A-{\mathrm{WENI}}_{i}+{∊}_{i}$$

(3) $$\mathrm{Pr}{\left(18.5<\mathrm{BMI}<25\right)}_{i}=\alpha_{1}+\beta_{1}A-{\mathrm{WENI}}_{i}+\delta_{i}$$

(4) $$\mathrm{Pr}{\left(\mathrm{MDD}=1\right)}_{i}=\alpha_{2}+\beta_{2}A-{\mathrm{WENI}}_{i}+\zeta_{i}$$

Our dependent variable is nutritional outcome, measured by BMI (continuous, binary status, indicating whether or not a person has a normal BMI, as well as its logarithmic transformation). Our focal explanatory variable is the A-WENI. All the regressions have robust standard errors that correct for heteroscedasticity. We also control for the physiological status of individuals, i.e., whether or not the WENI woman was pregnant at the time of interview, demographic group (relationship status, whether WENI woman, spouse, etc.) and age. The regression excludes village, household and individual level controls, because systematic differences in household socio-economic status (especially like wealth and education) in principle should reflect in the WENI variables themselves, adequately if not fully.

We find that A-WENI has significant positive association with BMI levels and minimum dietary diversity, indicating that higher values of nutritional empowerment are associated with better BMI (Table 5) and higher probability of the individual meeting the minimum dietary diversity norm. By this yardstick too the A-WENI is comparable with its parent index, the WENI.

**Table 5:** Relationship between nutritional empowerment and nutritional status using WENI survey data.

	WENI				A-WENI			
	BMI	Normal BMI (=1)	Log BMI	Minimum Dietary Diversity (=1)	BMI	Normal BMI (=1)	Log BMI	Minimum Dietary Diversity (=1)
Nutritionally Empowered (=1)	0.729***	0.277***	0.036***	0.324***	0.591***	0.272***	0.030***	0.388***
	(5.37)	(3.31)	(5.37)	(3.93)	(4.38)	(3.25)	(4.45)	(4.88)
								
State (Kerala = 1)	−0.212	−0.510***	−0.014	−0.532***	−0.308	−0.539***	−0.019*	−0.570***
	(−1.06)	(−4.02)	(−1.37)	(−6.09)	(−1.54)	(−4.30)	(−1.85)	(−6.56)
State (West Bengal = 1)	0.637***	−0.074	0.030***	−1.602***	0.531***	−0.091	0.025***	−1.605***
	(3.49)	(−0.53)	(3.36)	(−15.46)	(2.96)	(−0.67)	(2.85)	(−15.79)
State (Odisha = 1)	−1.008***	−0.687***	−0.050***	−2.467***	−1.137***	−0.709***	−0.056***	−2.460***
	(−5.23)	(−5.17)	(−5.21)	(−16.93)	(−6.03)	(−5.46)	(−5.99)	(−17.13)
State (Bihar = 1)	−1.018***	−0.644***	−0.050***	−2.404***	−1.150***	−0.667***	−0.057***	−2.408***
	(−5.72)	(−5.07)	(−5.68)	(−18.39)	(−6.61)	(−5.42)	(−6.55)	(−18.86)
Spouse (=1)	0.509***	0.291***	0.026***	0.025	0.553***	0.299***	0.028***	0.014
	(3.48)	(2.90)	(3.59)	(0.26)	(3.74)	(2.95)	(3.84)	(0.15)
MIL (=1)	0.160	0.034	0.009	0.221	0.206	0.060	0.011	0.257
	(0.50)	(0.18)	(0.53)	(1.33)	(0.63)	(0.30)	(0.67)	(1.55)
Older Woman	0.104	0.039	0.007	−0.284	0.140	0.065	0.008	−0.251
	(0.27)	(0.16)	(0.34)	(−1.42)	(0.36)	(0.26)	(0.43)	(−1.26)
Age (in completed years)	0.005	0.003	0.000	−0.006	0.003	0.002	0.000	−0.006
	(0.53)	(0.53)	(0.42)	(−1.17)	(0.36)	(0.40)	(0.25)	(−1.23)
Constant	20.321***	0.930***	3.006***	0.626***	20.534***	0.976***	3.016***	0.607***
	(69.19)	(4.75)	(202.22)	(3.63)	(70.34)	(5.04)	(204.47)	(3.67)
R-squared	0.115	–	0.112	–	0.110	–	0.107	–
Adj- R-squared	0.110	–	0.107	–	0.105	–	0.103	–
Chi-sq	–	106.044	–	808.189	–	106.011	–	798.982
N	1783	1783	1783	2342	1783	1783	1783	2342

Note:* significant at 10%, ** significant at 5%, *** significant at 1%. We control for different demographic groups by using dummies. t-statistic in parenthesis.

The predictive value of A-WENI is different for different nutritional outcomes mainly on account of how distal the nutritional outcomes are nutritional empowerment. For example, dietary diversity is much more sensitive to changes in circumstances that determine nutritional empowerment than is anemia or BMI. Under WENI, we treat dietary diversity as an intermediate outcome and BMI as one of the more distal final outcomes.^17^

## 4 Applying and Validating A-WENI in Maharashtra

Since the construction and validation of A-WENI is based on existing data from the WENI Survey, we validate the A-WENI in a new context using new survey data collected for this purpose from the western Indian state of Maharashtra. Using data from this new survey we validate the both the classification and prediction power of A-WENI. This serves two purposes. First, we wish to see whether the relationship between A-WENI and WENI as well as between A-WENI and nutritional outcomes holds outside of the data that was used to create it. Second, the western Indian state of Maharashtra offers a new context that likely differs significantly from the states that comprise the WENI Survey. Recall that the WENI survey had covered sites from states in northern (Bihar), eastern (Odisha, West Bengal) and southern India (Kerala and Tamil Nadu) – contexts that are significantly different from one another but also likely very different from Maharashtra.

### 4.1 The A-WENI Survey in Nashik, Maharashtra

To conduct the survey, we collaborated with Pragati Abhiyan, a rights-based civil society organization that works in Nashik district in Maharashtra. We surveyed 516 individuals in Nashik district, Maharashtra. The choice of the site was driven by the fact that the WENI had not been validated in the western part of India. The survey was held in February, 2020, and covered 13 villages spanning 5 administrative blocks in Nashik. We selected a mix of tribal and non-tribal villages to include communities with diverse social norms and resource constraints. The sample was selected to include diverse contexts and should therefore not be construed as representative of the region surveyed. As part of the survey, we interviewed young mothers with children below the age of five (209 individuals), a smaller sample of their male spouses and mother-in-laws (101 and 103 individuals respectively) and elder women above the age of 70 (103 individuals). This tablet-based survey was conducted in the local language (Marathi) and prior to the launching of the main survey we conducted extensive pretesting and pilot surveys to ensure that the questions were clearly framed, specific and understandable.

We use the same survey instruments as the WENI Survey, enabling us to compute both the original WENI with 33 indicators and the A-WENI with 20 indicators. We first compare these scores and the nutritional empowerment status based on these scores to verify that WENI and A-WENI produce broadly comparable results. We then use BMI and MDDS as measures of nutritional status, as with the earlier exercise using the WENI Survey. The BMI is a widely accepted measure that is easy to compute and appealing since it covers the entire spectrum of both under and over-nutrition.^18^

### 4.2 A-WENI validation using the Maharashtra A-WENI Survey

We validate A-WENI in the Maharashtra A-WENI Survey following the same process as for the WENI Survey. To evaluate A-WENI relative to WENI, we make the following comparisons. First, we conduct a rank order correlation test of the A-WENI and WENI scores using the Maharashtra A-WENI Survey data. We find that the rank order is preserved and Kendall’s tau-b score is 0.78, indicating high positive correlation. Second, we conduct a ROC analysis and find that 88.6% of the classifications into empowered or disempowered by the abridged WENI are correct (Appendix Table A2). Third, as with the earlier exercise using the WENI Survey, for the Maharashtra A-WENI Survey sample too we compute t-tests and proportion tests of nutritional outcomes based on empowerment status. We find that in both cases we mostly reject the null hypothesis of equality between the two groups, so that those who are assessed to be nutritionally empowered tend to have better nutritional status (Table 6).^19^

**Table 6:** Tests on nutritional outcomes (BMI) based on empowerment status in Maharashtra A-WENI Survey.

Test of Equality of Means – BMI					
	Disempowered		Empowered		P-value
	Mean	Obs	Mean	Obs	
WENI Woman	19.69	134	20.53	75	0.09
Spouse	20.30	23	22.07	77	0.04
MIL	20.36	60	22.40	43	0.02
Older Woman	19.73	88	23.06	15	0.00
All	19.88	305	21.66	210	0.00
					
Test of Equality of Proportions – of people with normal BMI					
WENI Woman	0.55	134	0.69	75	0.05
Spouse	0.70	23	0.82	77	0.21
MIL	0.60	60	0.81	43	0.02
Older Woman	0.53	88	0.93	15	0.00
All	0.57	305	0.78	210	0.00

Fourth, we estimate models using both BMI and Minimum Dietary Diversity(MDD) as outcome variables and the A-WENI as an explanatory variable. We estimate a least squares regression and a probit regression models (Eqs. 2,3 and 4) for the Maharashtra A-WENI sample, with robust standard errors that correct for heteroscedasticity. As with the earlier BMI regressions, we exclude the sample of overweight and obese individuals. We find that A-WENI remains a statistically significant predictor of nutritional outcomes for both the truncated and full samples. The strength and sign of the association between A-WENI and nutritional outcomes is broadly similar to that between WENI and nutritional outcomes. Higher values of A-WENI are thus associated with the statistically significantly higher values of nutritional outcomes (Table 7).^20^ In fact, for dietary diversity, the A-WENI has a significant positive association whereas WENI does not.^21^

**Table 7:** Relationship between nutritional empowerment and nutritional status in Maharashtra.

	WENI				A-WENI			
	BMI	Normal BMI (=1)	Log BMI	Minimum Dietary Diversity (=1)	BMI	Normal BMI (=1)	Log BMI	Minimum Dietary Diversity (=1)
Nutritionally empowered (=1)	0.972***	0.424***	0.050***	0.194	1.017***	0.471***	0.052***	0.265**
	(3.57)	(3.12)	(3.57)	(1.59)	(3.71)	(3.40)	(3.68)	(2.15)
								
Spouse (=1)	0.733**	0.248	0.038**	−0.129	0.679*	0.213	0.035*	−0.164
	(2.03)	(1.30)	(2.06)	(−0.76)	(1.88)	(1.10)	(1.92)	(−0.96)
MIL(=1)	−0.754	−0.487	−0.038	0.220	−0.721	−0.461	−0.036	0.240
	(−1.01)	(−1.30)	(−0.98)	(0.66)	(−0.96)	(−1.24)	(−0.92)	(0.72)
Older Woman (=1)	−1.402	−1.003*	−0.071	0.831	−1.320	−0.946	−0.067	0.879*
	(−1.19)	(−1.68)	(−1.16)	(1.56)	(−1.12)	(−1.59)	(−1.09)	(1.65)
Age (completed years)	0.026	0.021*	0.001	−0.009	0.023	0.019	0.001	−0.010
	(1.09)	(1.73)	(0.99)	(−0.80)	(0.96)	(1.60)	(0.86)	(−0.89)
Constant	18.260***	−0.511*	2.901***	0.096	18.395***	−0.454	2.908***	0.108
	(30.13)	(−1.65)	(92.09)	(0.34)	(31.15)	(−1.50)	(94.81)	(0.39)
R squared	0.071	–	0.072	–	0.073		0.073	
Adj. Rsquared	0.061	–	0.061	–	0.062		0.062	
Chi-sq	–	23.988	–	11.711		25.267		14.120
N	441	441	441	516	441	441	441	516

Note:* significant at 10%, ** significant at 5%, *** significant at 1%. We control for different demographic groups by using dummies. t-statistic in parenthesis.

These validation exercises offer confidence that relative to the 33-indicator WENI, the A-WENI does not produce statistically significantly different results either in terms of ranking individuals according to nutritional empowerment or in terms of predicting nutritional status. Collectively, they suggest that the A-WENI is a good catch-all measure for tracking nutritional empowerment that can replace WENI.

**Fig. 1:**
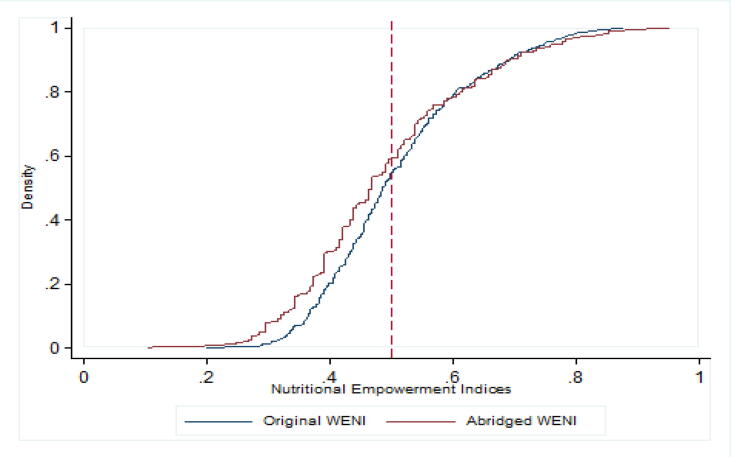
Cumulative Distribution Function of the Original (WENI) and Abridged WENI (A-WENI) in Maharashtra.

Despite the overwhelmingly encouraging results of the validation exercise, some cautionary notes are in order. Overall, the Maharashtra A-WENI results suggest that the A-WENI is more likely to deem someone as nutritionally empowered relative to WENI, given comparable cutoffs. However, this is not consistently the case. There may be some ranges of the nutritional empowerment scores where the opposite is true, where A-WENI deems a larger fraction to be nutritionally disempowered (Fig. 1). We note too that the proportion of those deemed to be nutritionally empowered by A-WENI can be different from those based on WENI for specific DDs (Table 8). These suggest that for a richer understanding of the impediments to empowerment, especially in terms of specific DDs, the original WENI may be more useful.

**Table 8:** Proportion of people empowered across Domain-Dimension in Maharashtra.

	WENI Women	Spouse	MIL	Older Women	A-WENI (ALL)	WENI (ALL)
Nutritional Empowerment	35.9	77.2	41.7	14.6	40.9	45.2
Food Knowledge	22.5	44.6	17.5	6.8	22.7	22.7
Food Resources	91.9	96	94.2	99	94.6	64.9
Food Agency	74.2	78.2	70.9	34	66.3	97.3
Health Knowledge	41.6	71.3	42.7	19.4	43.2	43.2
Health Resources	90.9	93.1	97.1	95.1	93.4	69.8
Health Agency	49.3	91.1	62.1	56.3	61.4	61.4
Institutions	40.7	94.1	58.3	24.3	51.4	87.8

## 5 Robustness checks

As evident from the discussion so far, we find persuasive evidence that the A-WENI based on LASSO methodology performs well relative to the original WENI in many ways. The A-WENI is able to classify individuals into empowered or disempowered categories with acceptable accuracy and is a good predictor of nutritional outcomes, like the original WENI. We perform a number of additional checks to boost the credibility of the A-WENI as a stand-alone measure of nutritional empowerment.

First, we generate a 20-indicator list using a different supervised machine learning algorithm known as Random Forest (Appendix Table A4). Even though it fulfills the criterion of representing at least 50% of the “ themes” in each DD, it is a poor predictor of minimum dietary diversity. We also used the elastic net method, fine tuning it by adjusting the penalty values to identify 20 indicators. The 20 indicators identified are the same as those we identify in A-WENI.

Second, thus far, our focus was on whether A-WENI and WENI both identify the same individuals as being nutritionally empowered based on replicating the underlying nutritional empowerment score (i.e. the WENI itself). We now test the ability of A-WENI to replicate directly the 0–1 nutritional empowerment status based on the WENI. This is to ensure that the A-WENI, beyond replicating the underlying variable on which the threshold is imposed, is also able to replicate the outcomes of these scores directly. For this, we treat the Y variable in Eq. 1 as nutritional empowerment status rather than empowerment scores. We find that the list of indicators generated using nutritional empowerment status is very similar to that generated by nutritional empowerment scores (Appendix Table A4). A detailed account of this is available in Anonymous.^22^

Third, we test the sensitivity of the A-WENI to thresholds of WENI, i.e., the nutritional empowerment scores, that forms the basis of classifying an individual as empowered (Appendix Table A5). We increase the threshold by 0.05 at a time and find that statistical significance of the coefficient of A-WENI remains statistically significant. We find that the threshold sensitivity is more robust when we use nutritional empowerment status as the predicted outcomes to construct A-WENI. This pattern is consistent with Narayanan et al. (2019) using the 33-indicator WENI. We find that using a LASSO to predict nutritional empowerment status rather than scores results in a marginally different A-WENI, but one is somewhat more robust to thresholds (Appendix Table A6).

Fourth, we also test whether we can identify an even smaller set of indicators, for example, just 10, to explore whether there is scope for further abbreviation (Appendix Table A2). We find that both the in-sample and out of sample RMSE is considerably higher than the 20-indicator A-WENI. A-WENI therefore appears to provide the best balance between the lowest RMSE and best prediction amongst all the cases.

Fifth, we switch the training and validation samples, using Bihar and Kerala as the training sites rather than Bihar and Odisha. This is to check if the choice of training samples matter. We find that it only matters in the margin. The 20 indicators identified are different from that of A-WENI by two indicators, both however belong to the respective DD of the indicator they replace.^23^

These procedures collectively suggest that the A-WENI is a credible alternative to the WENI. There are however some caveats to incorporating the A-WENI in a general purpose survey. First, the WENI has been constructed for rural south Asian contexts. It needs to be tested outside of South Asia, where machine learning could identify other indicators in new contexts. While some efforts are underway to validate the WENI is Sub-Saharan Africa, more work would be required to adapt the WENI to urban contexts. Second, there is a risk that incorporating the A-WENI in a general purpose survey might dilute the attention enumerators give to sensitive questions on intrahousehold issues and those such as domestic violence, that are critical for A-WENI. For this reason, if the A-WENI indicators are incorporated in a general purpose survey, enumerators need to be trained and advised appropriately since if indicators are missing in the construction of A-WENI, these need to be dropped from the analysis.

Third, while the A-WENI offers a quick snapshot that facilitates comparisons of nutritional empowerment across communities or socio-demographic groups, it has limited use for conducting nutritional empowerment diagnostics to identify key barriers that individuals face in achieving nutritional status in a meaningful way. This is because despite its predictive power and its ability to reproduce the ordering of individuals based on the nutritional empowerment scores, there is nevertheless a loss of detail, relative to the WENI, that might lead one to overlook some key actionable barriers to nutritional empowerment. As we pointed out in the previous section, the A-WENI does not replicate well empowerment status for specific DDs, even as it replicates overall nutritional empowerment well. Thus, using A-WENI for DD-specific analyses can potentially be misleading.

The A-WENI seems to trade away some of the 33-indicator-WENI’s ability to offer a granular perspective of the obstacles and barriers challenging women’s nutritional empowerment. To that extent, the A-WENI needs to be used cautiously.

## 6 Conclusion

The growing need to track and measure empowerment demands appropriate measures of empowerment that are easy to collect. A key challenge is that given its complex nature, there appears to be a trade-off between measuring empowerment comprehensively to reflect its many dimensions, which makes it expensive both in terms of time and cost to conduct a full-fledged survey to measure it, and to keep it simple and in the process dilute the rich conceptualizations we have of empowerment. We focus on WENI a recent measure of empowerment in the realm of nutrition in an effort to resolve this dilemma.

Our paper uses machine learning techniques that are data driven and transparent ways of reducing the number of indicators in an empowerment index in ways that reproduce, more or less, the outcomes of the parent index. Whereas this approach has been used in poverty measurement, this is perhaps a first application to the class of empowerment indices. We demonstrate its use to reduce and develop an abbreviated version of a recently created indicator of women’s empowerment in the realm of nutrition (the WENI).

The A-WENI consists of 20 indicators as opposed to the original 33. This reduction in number of indicators to a 20 indicator A-WENI, is our preferred recommendation as it covers at least 50% of the ‘themes’ originally selected for each DD, thus aligned closely to the spirit of the original WENI. While it is possible to reduce it even further to, say 15, as we explored, this latter leaves some dimensions with only one indicator, while performing more poorly relatively to A-WENI in its prediction of nutritional empowerment status.

We believe that the 20 indicators in A-WENI represent significant reductions in survey time (ranging from 20 to 30 min) while remaining faithful to the conceptual foundations underpinning WENI. The A-WENI’s 20 indicators are based on 28 distinct survey questions relative to the 59 required for the 33-indicator-WENI.

Furthermore, many of these 20 indicators likely already form part of any general purpose survey, so that the application of A-WENI perhaps demands the inclusion of just a few new questions to such surveys. Thus reducing the number of indicators from 33 to 20 reduces the survey time considerably and can easily be incorporated into a general purpose survey. Going from 20 to 15, in contrast, is unlikely to hold significant additional gains in terms of time or resources.

The A-WENI can serve to fill a crucial gap, especially in many developing country contexts. Very often household surveys do not capture information at a gender-disaggregated level and doing so can often be infeasible. Further, there has been a longstanding problem that most surveys tend to neglect issues relating to nutrition unless they are surveys specifically for health and nutrition, such as the Demographic Health Surveys (DHS). Consequently we know little about women’s lives and the barriers they face, especially in terms of their own nutritional well-being. Metrics such as the A-WENI can be leveraged easily to plug this gap – to secure key information that captures women’s empowerment in the realm of nutrition. Furthermore, its strong association with nutritional status precludes the need to collect anthropometric data, should there be serious resource or capacity constraints. The promise of A-WENI and the use of machine learning to help us better design measures of empowerment will be evident when these are tested and validated in other contexts outside of South Asia. This paper offers a way forward.

Developing a robust A-WENI can thus aid in an expansion of efforts to measure nutritional empowerment; this is key to understanding better the barriers and challenges women face and help identify ways in which women can improve their nutritional well-being in meaningful ways.

## CRediT authorship contribution statement

**Shree Saha & Sudha Narayanan:** Conceptualization, Methodology, Software, Data curation, Writing – original draft, Visualization, Investigation, Software, Validation, Writing – review & editing.

## Declaration of Competing Interest

The authors declare that they have no known competing financial interests or personal relationships that could have appeared to influence the work reported in this paper.

## Footnotes

1 The choice of the name is intended to parallel WEAI’s evolution and is analogous to A-WEAI – the abbreviated Women’s Empowerment in Agriculture Index.

2 While Kabeer (1999) defines empowerment in the dimensions of resource, agency, and achievements, where knowledge is a part of resources, to operationalize nutritional empowerment, Narayanan et al. (2019) considers knowledge as a separate dimension, given its importance in deciding the nutritional achievements.

3 While the WENI, an index based on this Grid, allows us to track progress in nutritional empowerment and is a good predictor of nutritional outcomes, the WEN Grid itself provides insights by domain-dimension (DD) which can be a helpful diagnostic tool in identifying areas that require policy action. For more on this, see (Narayanan et al., 2019).

4 WENI also introduces an additional domain of fertility for women in the reproductive age between 15 and 49, as it is a critical aspect of nutritional empowerment for young women. In this paper, we do not incorporate Fertility and focus instead on indicators that are relevant for all individuals.

5 At the DD level, a similar cutoff of 0.5 indicates whether an individual is empowered or disempowered in that specific DD.The choice of a cutoff is important. Too low a cutoff would make everyone empowered, rendering the exercise meaningless, just as too high a cutoff would leave everyone disempowered. One popular recommendation is to set the threshold to reflect aspirations, for example, by drawing on policy or programmatic goals (Alkire et al., 2015). We adopt 0.5 as an “aspirational” threshold that would likely clearly differentiate the different contexts within India on the basis of the WENI.

6 For young women in the reproductive age, the WENI uses an additional domain for Fertility with 10 more indicators (thus, a total of 43 indicators for WENI women). In this paper, however, we focus on generating A-WENI for any individual and therefore do not explicitly address the issue of fertility for younger women.

7 Although our goal was to reduce survey time, we do not explicitly include the time burden of items as a formal criterion in the process of abridging the WENI.

8 Schrider and Kern (2018) claims that even with simulation of data, machine learning produces models that are more robust to model misspecification in comparison to the traditional models.

9 In addition to LASSO, we also use the Random Forest algorithm and elastic LASSO to select indicators as a robustness check. Section 5 discusses this in detail.

12 Choosing 50% of the themes from each D-D as a guiding principle is somewhat subjective, driven by the need to combine a data-driven approach with one that does justice to the richness of a multidimensional index such as WENI.Our goal is to ensure that the A-WENI included at least one indicator for each DD. Our other goal is to maintain the ratio of the number of indicators across DD when we abridged the WENI, to maintain consistency. Ensuring that at least one indicator was chosen in the sparsest DD (which had just two indicators), equivalent to selecting 50% of the sparsest DD; this led to our goal of covering at least 50% of each DDs, in order to meet the second goal of maintaining the same ratio of indicators in A-WENI as in WENI. In theory, researchers could easily choose to include more reflecting their own preference, given the tradeoffs between richness and parsimony.

13 To illustrate our alternative for the 15-indicator exercise, we chose for all the three approaches to generate a fifteen indicator list to compute WENI and selecting penalty levels accordingly, Though the indices created based on these lists perform well in terms of validation (Table 3), they do not cover 50% of themes in each DD, diluting the conceptual basis for WENI and therefore not considered further.

14 All the four Information Criterion (AIC, AICC, BIC, EBIC) also generate the same 20 indicators as CVLasso and RLasso.

15 The sample for BMI as a continuous is restricted to those who are not overweight/obese(BMI<25), this is because we care about whether nutritional empowerment leads to less under-nutrition (Narayanan et al., 2019).

16 The MDD is based on FAO and FHI360 (2016) The minimum dietary diversity (MDD) is computed as 1 if the individual consumes at least 5 out of the ten food groups. For the MDD estimations, we use the full sample of individuals regardless of their BMI status.

17 Given that the samples we work with are not representative or large, the strength of these relationships needs to be interpreted with caution.

18 As mentioned earlier, we focus primarily on the under-weight and normal BMI i.e. BMI < 25, as we focus, as with WENI, on the link between nutritional empowerment and under-nutrition (Narayanan et al., 2019). However, the same results hold for the full sample as well.

19 In our alternate analysis where the LASSO predicts nutritional empowerment status, rather than scores, we find that the test of equality of means for WENI women is rejected at the 1% level.

20 The BMI distribution is right-skewed. We therefore transform the data and use logarithmic BMI values. We find that our results are robust to the transformation. We also implement group-wise regressions to analyze the predictive power of A-WENI for different sub-groups are available with the authors.Apart, from the robustness measures mentioned here, we also test if A-WENI, is good measure of empowerment for women in the fertility age group. This is important as decisions relating to fertility is a critical aspect of nutritional empowerment (Narayanan et al., 2019). We find that A-WENI is a good predictor of the nutritional empowerment status created including the fertility module and can therefore be used a measure of empowerment even for women belonging to the fertility age group (Appendix Table A3).

21 For MDD, we estimate the model on the full sample, including overweight/obese in the sample.

22 The new indicator list differs from the original by only two indicators. The indicators FAagrisay and Inoviolenceorsupport replace FAmajminsayent and Iparticipatedany in the list of indicators based on nutritional empowerment status. Although the factors are different, the new indicators belong to the same category of themes within their respective DDs as the indicators they replace.

23 Quite differently, beyond the scope of this work, we also implement the LASSO technique using nutritional outcomes, i.e., the BMI as a response variable rather than nutritional empowerment status, specifying selection of 20 indicators to make comparisons with the A-WENI set of indicators. We find that the overlap is significant and only a few indicators are different across the two sets (results available from the authors). In most of these cases, the indicators replaced belong to the same theme.

## Footnotes

10 The information criteria can be classified based on loss efficiency and selection efficiency. The former refers to the criterion that selects the smallest average squared error attainable by all candidate models. Model selection criterion on the other hand requires the true model to be selected with the probability tending to 1 as n tends to infinity. Thus, the former aims at prediction at and the latter at identification of true model. The AIC falls under the first category while the BIC in the latter. However, both of them perform poorly if the number of independent variables are more than the number of observations. To address this shortcoming, AICC and EBIC was constructed.

11 The two main parts of the rigorous lasso is the restricted eigenvalue condition and the penalization level. The first condition offers respite from the full rank condition required in OLS context, which can be a problem in case of high dimensionality of data. For the second condition, λ has to be large enough to control all noise in the data but at the same time has to be small enough to avoid shrinkage bias.
