# Supplementary material for: A simplified measure of nutritional empowerment: Using machine learning to abbreviate the Women’s Empowerment in Nutrition Index (WENI)
Source: World Dev. 2022 Jun;154:105860. doi: 10.1016/j.worlddev.2022.105860 (PMC8988240; doi:10.1016/j.worlddev.2022.105860)
Supplement: Supplementary file 1 [file mmc1.pdf]

Online Supplementary Materials

Appendix 1: Comparing A-WENI indicators with other Indian surveys

In general, with respect to Food and Health Knowledge (FK and HK), A-WENI explicitly asks whether the respondent is aware of how malaria is transmitted, the symptoms of anemia and use of ORS. The IHDS-2 includes some questions related to knowledge of health (reasons for occurrence of malaria, anemia and usage of ORS), but is somewhat different from A-WENI. While the A-WENI questionnaire explicitly inquires about the individual's knowledge of anemia, the IHDS-2 asks if the woman suffered from it during her last child-birth. The NHFS-4 does not ask explicitly about respondents knowledge of food and health. For example, NFHS-4 tests for knowledge of the presence of iodine in salt.

Both IHDS-2 and NFHS-4 have questions related to control of income/cash, access to land, permission to visit health facilities alone (or with permission). Both the surveys capture all these indicators, though some of these questions are posed differently from the WENI survey. Examples of these questions include, for example, whether the individual has freedom to visit bank/post-office or family. The IHDS-2 records freedom of movement without explicit mention of bank/post-office. Similarly while the IHDS-2 asks if the individual uses a phone for SMS/email, A-WENI explicitly asks if information related to government schemes are received through mobile phones. Even in terms of eating norms, while A-WENI asks every individual whether they eat last in the family, IHDS-2 has questions regarding the usual eating practices in the family, for example, whether everyone eats together or if men eat earlier than women. In contrast, this aspect is completely missing from the NFHS-4.

There are differences between the NFHS-4 and IHDS-2 as well. Like the NFHS-4, the IHDS-2 does not include questions regarding knowledge of calcium, iodine, but NFHS-4 also additionally lacks information regarding the knowledge of anemia amongst individuals. Additionally, the IHDS-2 does not enquire about the availability of assistance when individual is sick, or whether the individual receives information related to government schemes on their phones. The IHDS-2 also does not explicitly enquire about domestic violence at the respondent's home, but asks about the common practice in the village, likely because respondents might be reluctant to be candid in these matters. The WENI survey however, asks this information and explicitly specifically of all individuals.

The NFHS-4 on the other hand does not enquire about an individual's membership in any group (for example self-help groups, political or religious group etc.), or about the practice of veiling amongst women and the respondent's say in agricultural activities.

Table A1: Comparisons with IHDS-2 and NFHS-4 surveys

	A-WENI	IHDS -2	NFHS-4
1	FAassearnconsentown	Similar	X
2	FAcashcontrol	Similar	Similar
3	Famajminsayent	Similar	Similar
4	FKcalcium	X	X
5	FKiodine	X	X
6	FReatlast	Similar	X
7	FRland	Similar	Similar
8	HAalonefortreatment	Similar	Similar
9	HAhdecideownhealth	Similar	Similar
10	HAhealthvisitpermission	Similar	Similar
11	HKanemia	Similar	Similar
12	HKmalaria	Yes	Similar
13	HKors	Yes	Similar
14	HRdrinktoivent	Similar	Similar
15	HRhassistwhensick	X	Similar
16	Ianymemberownaccord	Similar	X
17	Idoveil	Similar	X
18	Ifreedommove	Similar	Similar
19	Imobileinformationgovt	Similar	Similar
20	Iparticipatedany	Similar	X

Table A2: Area under the ROC curve for Maharashtra

Indicator List	Observations	Area under the ROC curve	Standard error	Lower bound	Upper bound
Rlasso-15	516	0.853	0.015	0.823	0.882
CVLasso-15	516	0.847	0.016	0.816	0.878
Lasso with IC-15	516	0.847	0.016	0.816	0.878
20 Indicator List	516	0.886	0.014	0.859	0.914

Table A3: Relationship between the original empowerment status of WENI Woman (including the fertility module) with the A-WENI

	Empowerment status of WENI Woman in WENI survey	Empowerment status of WENI Woman in Maharashtra A-WENI Survey
A-WENI Nutritionally empowered (=1)	2.534***	2.332***
	(21.31)	(6.98)
Age (completed years)	-0.017*	-0.044
	(-1.95)	(-1.62)
Constant	-0.235	0.713
	(-0.91)	(1.08)
Chi-sq	454.175	48.704
N	1089.000	209.000

Note:* significant at 10%, ** significant at 5%, *** significant at 1%.

Table A4: List of Indicators based on different approaches

Sl. No.	20 Indicator List based on Nutritional Empowerment scores	15 Indicator List based on Nutritional Empowerment Indicator	10 Indicator list based on Nutritional Empowerment Status	20 Indicator list Based on Random Forest Algorithm	20 Indicator list with BMI as the Outcome Variable
1	FRland	FKcalcium	FKcalcium	FKiodine	FRpaidwork
2	FReatlast	FKiodine	FKiodine	FRjobandpds	FRland
3	FKcalcium	FAcashcontrol	HRdrinktoivent	FRselfemployment	FReatlast
4	FKiodine	HRhassistwhensick	HKanemia	FReatlast	FRselfemployment
5	FAassearnconsentown	HRdrinktoivent	HKors	FRpaidwork	FRfinsupportagrihhenter
6	FAagrisay	HKanemia	HKmalaria	FRfinsupportagrihhenter	FKcalcium
7	FAcashcontrol	HKors	HAalonefortreatment	FAdecisionpaidwrkbin	FAmajminsayent
8	HRhassistwhensick	HKmalaria	HAhealthvisitpermission	FAassearnconsentown	FAcashcontrol
9	HRdrinktoivent	HAalonefortreatment	HAhdecideownhealth	FAcashcontrol	HRdrinktoivent
10	HKanemia	HAhealthvisitpermission	Idoveil	HKors	HRriskinjhealth
11	HKors	HAhdecideownhealth		HKmalaria	HKanemia
12	HKmalaria	Ianymemberownaccord		HKanemia	HKors
13	HAalonefortreatment	Idoveil		HRintensityany	HKmalaria
14	HAhealthvisitpermission	Ifreedommove		HRdrinktoivent	HAalonefortreatment
15	HAhdecideownhealth	Iparticipatedany		HRhassistwhensick	HAhealthvisitpermission
16	Ianymemberownaccord			HAalonefortreatment	HAhdecideownhealth
17	Idoveil			HAhealthvisitpermission	Ianymemberownaccord
18	Imobileinformationgovt			HAhdecideownhealth	Idoveil
19	Ifreedommove			Ifreedommove	Inoviolenceorsupport
20	Inoviolenceorsupport			Idoveil	Iparticipatedany

Table A5: Relationship between nutritional outcomes and nutritional empowerment in A-WENI survey when predicted outcomes are nutritional empowerment scores)

	0.50			0.55			0.60			0.65		
	BMI	Normal BMI (=1)	MDD (=1)	BMI	Normal BMI (=1)	MDD (=1)	BMI	Normal BMI (=1)	MDD (=1)	BMI	Normal BMI (=1)	MDD (=1)
AWENI Nutritionally empowered (=1)	1.017***	0.471***	0.265**	1.261***	0.488***	0.302**	0.942***	0.435**	0.445***	0.549	0.235	0.280*
	(3.71)	(3.40)	(2.15)	(4.20)	(3.11)	(2.20)	(2.81)	(2.50)	(3.00)	(1.40)	(1.19)	(1.71)
Age	0.023	0.019	-0.010	0.017	0.018	-0.010	0.021	0.018	-0.011	0.027	0.021*	-0.009
	(0.96)	(1.60)	(-0.89)	(0.74)	(1.49)	(-0.96)	(0.87)	(1.52)	(-1.05)	(1.13)	(1.74)	(-0.83)
Spouse (=1)	0.679*	0.213	-0.164	0.613*	0.214	-0.178	0.780**	0.258	-0.212	0.911**	0.322*	-0.147
	(1.88)	(1.10)	(-0.96)	(1.68)	(1.11)	(-1.03)	(2.13)	(1.36)	(-1.23)	(2.48)	(1.70)	(-0.86)
MIL (=1)	-0.721	-0.461	0.240	-0.608	-0.435	0.248	-0.636	-0.426	0.275	-0.822	-0.501	0.219
	(-0.96)	(-1.24)	(0.72)	(-0.82)	(-1.17)	(0.75)	(-0.83)	(-1.13)	(0.83)	(-1.07)	(-1.35)	(0.66)
Older Woman (=1)	-1.320	-0.946	0.879*	-1.078	-0.906	0.904*	-1.346	-0.962	0.953*	-1.718	-1.115*	0.807
	(-1.12)	(-1.59)	(1.65)	(-0.91)	(-1.52)	(1.69)	(-1.11)	(-1.60)	(1.78)	(-1.43)	(-1.87)	(1.51)
Constant	18.395***	-0.454	0.108	18.600***	-0.362	0.159	18.639***	-0.342	0.181	18.581***	-0.361	0.162
	(31.15)	(-1.50)	(0.39)	(31.98)	(-1.20)	(0.58)	(31.25)	(-1.12)	(0.66)	(30.97)	(-1.19)	(0.58)
N	441	441	516	441	441	516	441	441	516	441	441	516
R-Sq	0.073	-	-	0.080	-	-	0.061	-	-	0.049	-	-
Adj R-sq	0.062	-	-	0.069	-	-	0.050	-	-	0.038	-	-
Chi sq	-	25.267	14.120	-	22.561	13.761	-	18.763	17.719	-	15.332	11.590

Note:* significant at 10%, ** significant at 5%, *** significant at 1%. t-statistic in parenthesis.

Table A5 (contd.): Relationship between nutritional outcomes and nutritional empowerment in A-WENI survey when predicted outcomes are nutritional empowerment scores)

	0.70			0.75			0.80			0.85		
	BMI	Normal BMI (=1)	MDD (=1)	BMI	Normal BMI (=1)	MDD (=1)	BMI	Normal BMI (=1)	MDD (=1)	BMI	Normal BMI (=1)	MDD (=1)
AWENI Nutritionally empowered (=1)	1.205**	0.561**	0.170	1.342***	0.668*	0.130	1.220**	0.966*	0.205	0.859	0.000	-0.239
	(2.57)	(2.10)	(0.88)	(2.80)	(1.89)	(0.54)	(2.04)	(1.87)	(0.65)	(1.39)	(.)	(-0.59)
Age	0.026	0.020*	-0.008	0.029	0.021*	-0.008	0.028	0.021*	-0.008	0.030	0.022*	-0.008
	(1.06)	(1.70)	(-0.77)	(1.22)	(1.80)	(-0.74)	(1.18)	(1.77)	(-0.74)	(1.25)	(1.81)	(-0.73)
Spouse (=1)	0.895**	0.311*	-0.096	0.881**	0.312*	-0.087	0.969***	0.345*	-0.084	0.972***	0.313*	-0.038
	(2.52)	(1.66)	(-0.57)	(2.46)	(1.66)	(-0.51)	(2.68)	(1.84)	(-0.50)	(2.62)	(1.66)	(-0.22)
MIL (=1)	-0.785	-0.492	0.213	-0.868	-0.520	0.211	-0.851	-0.516	0.211	-0.884	-0.517	0.208
	(-1.02)	(-1.31)	(0.64)	(-1.13)	(-1.40)	(0.64)	(-1.11)	(-1.39)	(0.64)	(-1.15)	(-1.39)	(0.63)
Older Woman (=1)	-1.631	-1.089*	0.764	-1.816	-1.149*	0.746	-1.806	-1.138*	0.745	-1.901	-1.172**	0.735
	(-1.34)	(-1.82)	(1.44)	(-1.52)	(-1.94)	(1.41)	(-1.51)	(-1.92)	(1.41)	(-1.59)	(-1.98)	(1.39)
Constant	18.598***	-0.358	0.162	18.554***	-0.368	0.161	18.594***	-0.354	0.161	18.574***	-0.354	0.161
	(30.46)	(-1.17)	(0.59)	(31.11)	(-1.21)	(0.58)	(31.13)	(-1.17)	(0.59)	(31.07)	(-1.17)	(0.59)
N	441	441	516	441	441	516	441	441	516	441	434	516
R-Sq	0.059	-	-	0.056	-	-	0.050	-	-	0.047	-	-
Adj R-sq	0.048	-	-	0.045	-	-	0.039	-	-	0.036	-	-
Chi sq	-	16.494	9.619	-	16.355	9.150	-	19.075	9.289	-	11.575	9.259

Note:* significant at 10%, ** significant at 5%, *** significant at 1%. t-statistic in parenthesis.

Table A6: Relationship between nutritional outcomes and nutritional empowerment in A-WENI survey when predicted outcomes are nutritional empowerment status)

	0.50			0.55			0.60			0.65		
	BMI	Normal BMI (=1)	MDD (=1)	BMI	Normal BMI (=1)	MDD (=1)	BMI	Normal BMI (=1)	MDD (=1)	BMI	Normal BMI (=1)	MDD (=1)
A-WENI Nutritionally empowered (=1)	0.993***	0.386***	0.280**	0.880***	0.427***	0.267**	0.866***	0.371**	0.340**	0.788**	0.367**	0.446***
	(-3.73)	(-2.94)	(-2.3)	(-3.11)	(-3.02)	(-2.13)	(-2.79)	(-2.3)	(-2.41)	(-2.25)	(-2.03)	(-2.91)
Age (completed years)	0.028	0.022*	-0.009	0.023	0.019	-0.009	0.02	0.018	-0.011	0.023	0.019	-0.011
	(-1.18)	(-1.8)	(-0.81)	(-0.99)	(-1.62)	(-0.88)	(-0.85)	(-1.52)	(-1.01)	(-0.96)	(-1.6)	(-1.02)
Spouse(=1)	0.715**	0.249	-0.157	0.713*	0.22	-0.171	0.782**	0.269	-0.185	0.826**	0.279	-0.208
	(-1.97)	(-1.3)	(-0.93)	-1.93	-1.14	(-0.99)	-2.17	-1.41	(-1.07)	-2.25	-1.46	(-1.20)
MIL(=1)	-0.887	-0.54	0.204	-0.735	-0.463	0.233	-0.623	-0.425	0.273	-0.68	-0.446	0.28
	(-1.17)	(-1.44)	(-0.62)	(-0.97)	(-1.24)	-0.7	(-0.82)	(-1.13)	-0.82	(-0.89)	(-1.18)	-0.84
Older Woman (=1)	-1.511	-1.060*	0.859	-1.423	-0.98	0.861	-1.297	-0.953	0.930*	-1.484	-1.021*	0.930*
	(-1.29)	(-1.78)	(-1.63)	(-1.19)	(-1.64)	-1.62	(-1.07)	(-1.58)	-1.74	(-1.23)	(-1.69)	-1.73
Constant	18.129***	-0.544*	0.037	18.457***	-0.427	0.116	18.632***	-0.345	0.171	18.623***	-0.35	0.179
	(-29.55)	(-1.75)	(-0.13)	(-31.27)	(-1.41)	(-0.42)	(-31.52)	(-1.14)	(-0.62)	(-31.19)	(-1.15)	(-0.65)
R-squared	0.073	-	-	0.065	-	-	0.06	-	-	0.055	-	-
Adjusted R-squared	0.063	-	-	0.054	-	-	0.05	-	-	0.045	-	-
Chi-sq	-	22.481	14.844	-	22.708	13.914	-	18.838	14.746	-	17.629	17.119
N	441	441	516	441	441	516	441	441	516	441	441	516

Note:* significant at 10%, ** significant at 5%, *** significant at 1%.

Table A6 (contd.): Relationship between nutritional outcomes and nutritional empowerment in A-WENI survey when predicted outcomes are nutritional empowerment status)

	0.70			0.75			0.80			0.85		
	BMI	Normal BMI (=1)	MDD (=1)	BMI	Normal BMI (=1)	MDD (=1)	BMI	Normal BMI (=1)	MDD (=1)	BMI	Normal BMI (=1)	MDD (=1)
A-WENI Nutritionally empowered (=1)	0.785*	0.288	0.339*	0.961*	0.44	0.23	1.419***	1.177**	0.234	1.406*	0	0.026
	(-1.87)	(-1.33)	(-1.93)	(-1.88)	(-1.46)	(-1.04)	(-2.93)	(-2.38)	(-0.87)	(-1.87)	(.)	(-0.07)
Age (completed years)	0.029	0.022*	-0.008	0.027	0.021*	-0.009	0.028	0.021*	-0.008	0.028	0.020*	-0.008
	(-1.2)	(-1.82)	(-0.74)	(-1.1)	(-1.75)	(-0.80)	(-1.16)	(-1.76)	(-0.76)	(-1.16)	(-1.71)	(-0.73)
Spouse(=1)	0.892**	0.321*	-0.149	0.955***	0.335*	-0.096	0.964***	0.347*	-0.09	0.986***	0.351*	-0.065
	(-2.45)	(-1.71)	(-0.87)	(-2.67)	(-1.8)	(-0.58)	(-2.7)	(-1.85)	(-0.54)	(-2.74)	(-1.86)	(-0.39)
MIL(=1)	-0.86	-0.524	0.203	-0.803	-0.506	0.226	-0.816	-0.499	0.216	-0.827	-0.484	0.208
	(-1.12)	(-1.40)	-0.61	(-1.03)	(-1.35)	-0.68	(-1.06)	(-1.34)	-0.65	(-1.08)	(-1.30)	-0.63
Older Woman (=1)	-1.791	-1.161*	0.764	-1.712	-1.133*	0.78	-1.756	-1.111*	0.755	-1.784	-1.103*	0.735
	(-1.48)	(-1.95)	-1.43	(-1.40)	(-1.88)	-1.46	(-1.47)	(-1.88)	-1.43	(-1.49)	(-1.86)	-1.39
Constant	18.525***	-0.384	0.139	18.603***	-0.361	0.17	18.582***	-0.362	0.161	18.612***	-0.335	0.16
	(-30.62)	(-1.26)	(-0.5)	(-30.51)	(-1.18)	(-0.61)	(-31.15)	(-1.20)	(-0.59)	(-31.09)	(-1.11)	(-0.58)
R-squared	0.052	-	-	0.052	-	-	0.054	-	-	0.05	-	-
Adjusted R-squared	0.042	-	-	0.042	-	-	0.044	-	-	0.039	-	-
Chi-sq	-	15.279	12.354	-	15.381	9.921	-	20.795	9.62	-	12.184	8.9
N	441	441	516	441	441	516	441	441	516	441	433	516

Note:* significant at 10%, ** significant at 5%, *** significant at 1%.
